# 3-{[1-(2,3,5-Tri-*O*-benzoyl-β-d-ribofur­an­os-1-yl)-1*H*-1,2,3-triazol-4-yl]meth­yl}quin­a­zolin-4(3*H*)-one

**DOI:** 10.1107/S1600536812042778

**Published:** 2012-10-20

**Authors:** Abdelaaziz Ouahrouch, Moha Taourirte, Mohamed El Azhari, Mohamed Saadi, Lahcen El Ammari

**Affiliations:** a Laboratoire de Chimie Bio-organique et Macromoléculaire, Faculté des Sciences et Techniques Guéliz, Marrakech, Morocco; b Laboratoire de la Matière Condensée et des Nanostructures, Faculté des Sciences et Techniques Guéliz, Marrakech, Morocco; c Laboratoire de Chimie du Solide Appliquée, Faculté des Sciences, Université Mohammed V-Agdal, Avenue Ibn Battouta, BP 1014, Rabat, Morocco

## Abstract

In the compound, C_37_H_29_N_5_O_8_, the quinazoline residue forms a dihedral angle of 72.90 (9)° with the triazole ring. The furan ring adopts a twist conformation. In the crystal, the mol­ecules are linked by non-classical C—H⋯N and C—H⋯O hydrogen bonds, building an infinite three-dimensional network.

## Related literature

For details of the synthesis, see: Ines *et al.* (2008 ▶); Krim *et al.* (2009 ▶); Huisgen (1963 ▶), Wu *et al.* (2004 ▶). For background to the biological activity of quinazolines, see: Traxler (1998 ▶); Bridges (2001 ▶); Wakeling (2005 ▶); Diana & Nitz (1993 ▶); Chen *et al.* (2000 ▶); Manfredini *et al.* (2000 ▶). For conformational analysis, see: Cremer & Pople (1975 ▶).
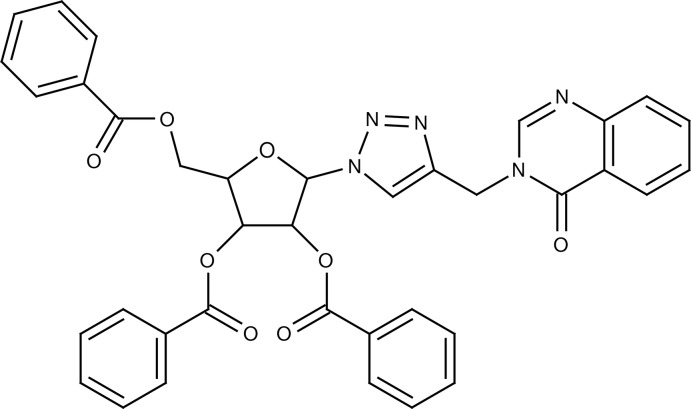


## Experimental

### 

#### Crystal data

C_37_H_29_N_5_O_8_
*M* *_r_* = 671.65
Monoclinic, 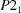

*a* = 11.2646 (2) Å
*b* = 5.6471 (1) Å
*c* = 25.7507 (4) Å
β = 99.595 (1)°
*V* = 1615.15 (5) Å^3^
*Z* = 2
Mo *K*α radiation
μ = 0.10 mm^−1^
*T* = 296 K
0.33 × 0.29 × 0.25 mm

#### Data collection

Bruker X8 APEXII diffractometer
26180 measured reflections
4490 independent reflections
3651 reflections with *I* > 2σ(*I*)
*R*_int_ = 0.029

#### Refinement

*R*[*F* ^2^ > 2σ(*F* ^2^)] = 0.033
*wR*(*F* ^2^) = 0.081
*S* = 1.03
4490 reflections
451 parameters
1 restraint
H-atom parameters constrained
Δρ_max_ = 0.14 e Å^−3^
Δρ_min_ = −0.13 e Å^−3^

### 

Data collection: *APEX2* (Bruker, 2005 ▶); cell refinement: *SAINT* (Bruker, 2005 ▶); data reduction: *SAINT*; program(s) used to solve structure: *SHELXS97* (Sheldrick, 2008 ▶); program(s) used to refine structure: *SHELXL97* (Sheldrick, 2008 ▶); molecular graphics: *ORTEP-3 for Windows* (Farrugia,1997 ▶); software used to prepare material for publication: *PLATON* (Spek, 2009 ▶) and *publCIF* (Westrip, 2010 ▶).

## Supplementary Material

Crystal structure: contains datablock(s) I, global. DOI: 10.1107/S1600536812042778/bt6845sup1.cif

Structure factors: contains datablock(s) I. DOI: 10.1107/S1600536812042778/bt6845Isup2.hkl

Additional supplementary materials:  crystallographic information; 3D view; checkCIF report

## Figures and Tables

**Table 1:** Hydrogen-bond geometry (Å, °)

*D*—H⋯*A*	*D*—H	H⋯*A*	*D*⋯*A*	*D*—H⋯*A*
C3—H3⋯O6^i^	0.93	2.57	3.483 (3)	168
C13—H13⋯O6^ii^	0.98	2.36	3.293 (3)	159
C6—H6⋯N1^iii^	0.93	2.62	3.390 (3)	141

Symmetry codes: (i) 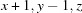; (ii) 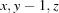; (iii) 
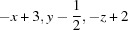
.

**Fig. 1.**
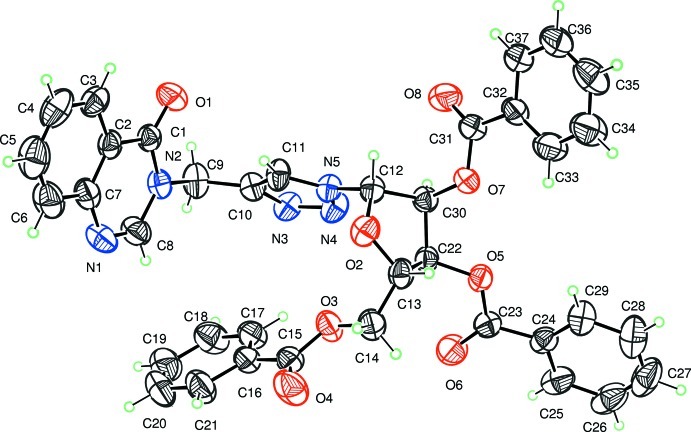
Molecular structure of the title compound with the atom-labelling scheme. Displacement ellipsoids are drawn at the 50% probability level. H atoms are represented as small circles.

## supplementary crystallographic information

### Comment

Quinazoline and its derivatives are an interesting class of heterocyclic compounds that have drawn much attention because of their biological and pharmaceutical activities including a wide range of antitumor activity (Traxler, 1998; Bridges, 2001; Wakeling, 2005). Furthermore, triazole heterocycles are potent antiviral (Diana & Nitz, 1993), antimicrobial (Chen *et al.*, 2000), and anti-proliferate agents (Manfredini *et al.*, 2000). The pharmaceutical importance of triazoles has prompted the design and synthesis of various triazolonucleosides.

The most interesting method for the synthesis of triazoles is the Huisgen 1,3-dipolar cycloaddition of organic azides with alkynes (Huisgen, 1963). Copper-catalyzed click chemistry is an efficient method that uses azides and terminal acetylenes to have 1,4-disubstituted-1,2,3- triazole products with excellent selectivity and high yield (Wu *et al.*, 2004). In connection to our studies on the synthesis of new nucleosides, we decided to explore the feasibility of the 'click' chemistry for the synthesis of novel 1,2,3-triazoles containing the quinazolinone moiety.

The molecule of the title compound contains a quinazolinone moiety linked to a furan ring through a triazole ring. As shown in Fig.1, the furan ring is also connected to three benzoyl rings. The furan ring which adopts a twist conformation as indicated by Cremer & Pople (1975) puckering parameters Q(2)= 0.365 (2) Å and the phase angle φ = 85.7 (3)°. The five-membered ring (O2 C12 C13 C22 C30) is nearly perpendicular to (N3 N4 N5 C10 C11), (C16 to C21), (C24 to C29) and to (C32 to C37) with the dihedral angles of 79.6 (2), 75.9 (2), 83.4 (2) and 88.2 (2)°, respectively.

An intermolecular C–H···N and C–H···O non classic hydrogen bonds, building an infinite three-dimensional network ensure the cohesion of the crystal structure (see Table 1).

### Experimental

The title compound, 3-((2,3,5-tri-*O*-benzoyl-β-*D*-ribofuranosyl- 1*H*-1,2,3-τriazol-4-yl)methyl) quinazolin-4(3*H*)-one was achieved by cycloaddition of propargylated quinazolinone with 2,3,5-tri-*O*-benzoyl-β-D– ribofuranosyl azide under microwave conditions with CuI as catalyst and without solvent. The product was obtained with quantitative yield (84%) and short reaction time (Ines *et al.* (2008); Krim *et al.* (2009)). The crude product was purified passing through a column packed with silica gel. Crystal suitable for X-ray analysis was obtained by slow evaporation of a methanol.

### Refinement

H atoms were located in a difference map and treated as riding with C—H = 0.97 Å and 0.93 Å for –CH_2_– and aromatic CH, respectively and with *U*_iso_(H) = 1.2 *U*_eq_(C). In the absence of significant anomalous scattering, the absolute configuration could not be reliably determined and any references to the Flack parameter were removed.

### Crystal data

**Table:**

C_37_H_29_N_5_O_8_	*F*(000) = 700
*M**_r_* = 671.65	*D*_x_ = 1.381 Mg m^−^^3^
Monoclinic, *P*2_1_	Melting point: 454 K
Hall symbol: P 2yb	Mo *K*α radiation, λ = 0.71073 Å
*a* = 11.2646 (2) Å	Cell parameters from 4490 reflections
*b* = 5.6471 (1) Å	θ = 0.8–28.5°
*c* = 25.7507 (4) Å	µ = 0.10 mm^−^^1^
β = 99.595 (1)°	*T* = 296 K
*V* = 1615.15 (5) Å^3^	Block, colourless
*Z* = 2	0.33 × 0.29 × 0.25 mm

### Data collection

**Table:**

Bruker X8 APEXII diffractometer	3651 reflections with *I* > 2σ(*I*)
Radiation source: fine-focus sealed tube	*R*_int_ = 0.029
Graphite monochromator	θ_max_ = 28.5°, θ_min_ = 0.8°
φ and ω scans	*h* = −15→13
26180 measured reflections	*k* = −6→7
4490 independent reflections	*l* = −32→34

### Refinement

**Table:**

Refinement on *F*^2^	Primary atom site location: structure-invariant direct methods
Least-squares matrix: full	Secondary atom site location: difference Fourier map
*R*[*F*^2^ > 2σ(*F*^2^)] = 0.033	Hydrogen site location: difference Fourier map
*wR*(*F*^2^) = 0.081	H-atom parameters constrained
*S* = 1.03	*w* = 1/[σ^2^(*F*_o_^2^) + (0.0384*P*)^2^ + 0.1513*P*] where *P* = (*F*_o_^2^ + 2*F*_c_^2^)/3
4490 reflections	(Δ/σ)_max_ = 0.001
451 parameters	Δρ_max_ = 0.14 e Å^−^^3^
1 restraint	Δρ_min_ = −0.13 e Å^−^^3^

### Special details

**Table:**

Geometry. All s.u.'s (except the s.u. in the dihedral angle between two l.s. planes) are estimated using the full covariance matrix. The cell s.u.'s are taken into account individually in the estimation of s.u.'s in distances, angles and torsion angles; correlations between s.u.'s in cell parameters are only used when they are defined by crystal symmetry. An approximate (isotropic) treatment of cell s.u.'s is used for estimating s.u.'s involving l.s. planes.
Refinement. Refinement of *F*^2^ against all reflections. The weighted *R*-factor *wR* and goodness of fit *S* are based on *F*^2^, conventional *R*-factors *R* are based on *F*, with *F* set to zero for negative *F*^2^. The threshold expression of *F*^2^ > 2σ(*F*^2^) is used only for calculating *R*-factors(gt) *etc*. and is not relevant to the choice of reflections for refinement. *R*-factors based on *F*^2^ are statistically about twice as large as those based on *F*, and *R*- factors based on all data will be even larger.

### Fractional atomic coordinates and isotropic or equivalent isotropic displacement parameters (Å^2^)

**Table:**

	*x*	*y*	*z*	*U*_iso_*/*U*_eq_	
O1	1.48779 (13)	0.5193 (3)	0.77079 (5)	0.0599 (4)	
C1	1.47437 (15)	0.5402 (3)	0.81663 (7)	0.0394 (4)	
C2	1.52690 (15)	0.3864 (4)	0.85969 (7)	0.0409 (4)	
C3	1.60106 (17)	0.1978 (4)	0.85044 (9)	0.0527 (5)	
H3	1.6160	0.1675	0.8166	0.063*	
C4	1.6516 (2)	0.0575 (5)	0.89200 (12)	0.0715 (7)	
H4	1.7013	−0.0682	0.8863	0.086*	
C5	1.6288 (2)	0.1029 (6)	0.94223 (11)	0.0803 (8)	
H5	1.6638	0.0073	0.9700	0.096*	
C6	1.5565 (2)	0.2845 (6)	0.95168 (9)	0.0702 (7)	
H6	1.5421	0.3122	0.9857	0.084*	
C7	1.50357 (18)	0.4298 (4)	0.91030 (8)	0.0491 (5)	
N1	1.42881 (18)	0.6125 (4)	0.92126 (7)	0.0596 (5)	
N2	1.40433 (13)	0.7224 (3)	0.83175 (6)	0.0419 (3)	
C8	1.38486 (19)	0.7462 (4)	0.88269 (8)	0.0545 (5)	
H8	1.3355	0.8701	0.8898	0.065*	
C9	1.35481 (18)	0.8972 (4)	0.79210 (8)	0.0517 (5)	
H9A	1.4119	0.9228	0.7683	0.062*	
H9B	1.3444	1.0464	0.8095	0.062*	
C10	1.23629 (16)	0.8228 (3)	0.76079 (7)	0.0407 (4)	
C11	1.18997 (17)	0.6052 (4)	0.74762 (8)	0.0445 (4)	
H11	1.2253	0.4588	0.7566	0.053*	
N3	1.15628 (14)	0.9885 (3)	0.73967 (6)	0.0439 (4)	
N4	1.06111 (14)	0.8827 (3)	0.71381 (7)	0.0456 (4)	
N5	1.08115 (13)	0.6486 (3)	0.71851 (6)	0.0392 (3)	
C12	0.98816 (16)	0.4739 (3)	0.69944 (7)	0.0396 (4)	
H12	1.0233	0.3450	0.6815	0.048*	
O2	0.93768 (11)	0.3821 (3)	0.74159 (5)	0.0486 (3)	
C13	0.81906 (16)	0.4779 (4)	0.74176 (7)	0.0441 (4)	
H13	0.7602	0.3536	0.7298	0.053*	
C14	0.80732 (18)	0.5416 (5)	0.79708 (8)	0.0529 (5)	
H14A	0.7273	0.6033	0.7979	0.063*	
H14B	0.8185	0.4013	0.8191	0.063*	
C15	0.92262 (17)	0.7284 (4)	0.87000 (7)	0.0495 (5)	
O3	0.89576 (12)	0.7166 (3)	0.81724 (5)	0.0523 (4)	
O4	0.87382 (16)	0.6050 (4)	0.89822 (6)	0.0759 (5)	
C16	1.01456 (17)	0.9096 (4)	0.88883 (7)	0.0482 (5)	
C17	1.05099 (19)	1.0778 (4)	0.85589 (8)	0.0537 (5)	
H17	1.0199	1.0759	0.8201	0.064*	
C18	1.1337 (2)	1.2490 (5)	0.87610 (10)	0.0701 (7)	
H18	1.1572	1.3638	0.8540	0.084*	
C19	1.1811 (2)	1.2495 (6)	0.92904 (10)	0.0770 (8)	
H19	1.2372	1.3641	0.9425	0.092*	
C20	1.1460 (3)	1.0813 (7)	0.96195 (10)	0.0810 (8)	
H20	1.1786	1.0821	0.9976	0.097*	
C21	1.0629 (2)	0.9123 (6)	0.94236 (8)	0.0688 (7)	
H21	1.0389	0.7995	0.9648	0.083*	
C22	0.80514 (15)	0.6720 (3)	0.70008 (7)	0.0388 (4)	
H22	0.8349	0.8231	0.7158	0.047*	
O5	0.68225 (11)	0.6958 (2)	0.67425 (5)	0.0454 (3)	
O6	0.65640 (13)	0.9904 (3)	0.72934 (6)	0.0625 (4)	
C23	0.61705 (16)	0.8669 (4)	0.69278 (7)	0.0420 (4)	
C24	0.49230 (16)	0.8815 (4)	0.66316 (7)	0.0440 (4)	
C25	0.4252 (2)	1.0813 (5)	0.67048 (9)	0.0592 (6)	
H25	0.4599	1.2044	0.6918	0.071*	
C26	0.3065 (2)	1.0963 (6)	0.64598 (11)	0.0767 (9)	
H26	0.2615	1.2304	0.6506	0.092*	
C27	0.2555 (2)	0.9163 (7)	0.61522 (12)	0.0833 (10)	
H27	0.1751	0.9266	0.5995	0.100*	
C28	0.3214 (2)	0.7190 (7)	0.60707 (11)	0.0827 (9)	
H28	0.2860	0.5976	0.5855	0.099*	
C29	0.4405 (2)	0.7013 (5)	0.63111 (9)	0.0620 (6)	
H29	0.4855	0.5682	0.6256	0.074*	
C30	0.88453 (15)	0.5871 (4)	0.66172 (7)	0.0400 (4)	
H30	0.9110	0.7159	0.6408	0.048*	
O7	0.82166 (11)	0.4025 (3)	0.62974 (5)	0.0452 (3)	
O8	0.97436 (13)	0.3887 (4)	0.58366 (6)	0.0704 (5)	
C31	0.87760 (18)	0.3172 (4)	0.59115 (8)	0.0494 (5)	
C32	0.80817 (18)	0.1250 (4)	0.56099 (7)	0.0486 (5)	
C33	0.6908 (2)	0.0733 (5)	0.56586 (9)	0.0645 (6)	
H33	0.6516	0.1623	0.5883	0.077*	
C34	0.6317 (3)	−0.1120 (6)	0.53723 (10)	0.0793 (8)	
H34	0.5524	−0.1467	0.5402	0.095*	
C35	0.6900 (3)	−0.2449 (6)	0.50442 (10)	0.0794 (8)	
H35	0.6503	−0.3700	0.4854	0.095*	
C36	0.8059 (3)	−0.1932 (6)	0.49975 (10)	0.0810 (9)	
H36	0.8450	−0.2836	0.4776	0.097*	
C37	0.8650 (2)	−0.0097 (6)	0.52735 (9)	0.0666 (7)	
H37	0.9438	0.0251	0.5236	0.080*	

### Atomic displacement parameters (Å^2^)

**Table:**

	*U*^11^	*U*^22^	*U*^33^	*U*^12^	*U*^13^	*U*^23^
O1	0.0670 (9)	0.0774 (11)	0.0363 (7)	0.0054 (9)	0.0121 (6)	0.0024 (7)
C1	0.0354 (9)	0.0417 (10)	0.0403 (9)	−0.0081 (8)	0.0036 (7)	−0.0004 (8)
C2	0.0346 (8)	0.0445 (10)	0.0420 (9)	−0.0090 (8)	0.0021 (7)	0.0018 (8)
C3	0.0383 (10)	0.0541 (13)	0.0657 (13)	−0.0055 (9)	0.0085 (9)	−0.0017 (11)
C4	0.0495 (12)	0.0572 (15)	0.105 (2)	0.0085 (11)	0.0051 (12)	0.0176 (15)
C5	0.0767 (16)	0.082 (2)	0.0746 (17)	0.0064 (16)	−0.0094 (13)	0.0320 (16)
C6	0.0818 (16)	0.0797 (18)	0.0457 (12)	−0.0010 (15)	0.0006 (11)	0.0169 (13)
C7	0.0509 (11)	0.0548 (13)	0.0394 (10)	−0.0091 (10)	0.0012 (8)	0.0036 (9)
N1	0.0744 (12)	0.0659 (12)	0.0388 (9)	0.0013 (11)	0.0105 (8)	−0.0050 (9)
N2	0.0447 (8)	0.0390 (8)	0.0394 (8)	−0.0056 (7)	−0.0005 (6)	0.0015 (7)
C8	0.0628 (12)	0.0528 (13)	0.0472 (11)	0.0010 (11)	0.0075 (9)	−0.0084 (10)
C9	0.0548 (11)	0.0391 (11)	0.0561 (12)	−0.0088 (10)	−0.0059 (9)	0.0077 (9)
C10	0.0464 (10)	0.0327 (10)	0.0417 (10)	−0.0007 (8)	0.0032 (8)	0.0027 (8)
C11	0.0437 (10)	0.0319 (9)	0.0529 (11)	0.0057 (8)	−0.0068 (8)	0.0032 (9)
N3	0.0439 (8)	0.0296 (7)	0.0580 (10)	0.0008 (7)	0.0074 (7)	0.0036 (7)
N4	0.0409 (8)	0.0308 (8)	0.0638 (10)	0.0030 (7)	0.0050 (7)	0.0048 (8)
N5	0.0400 (8)	0.0290 (8)	0.0467 (8)	0.0026 (6)	0.0018 (6)	0.0008 (6)
C12	0.0410 (9)	0.0318 (9)	0.0445 (10)	0.0010 (8)	0.0027 (7)	−0.0018 (8)
O2	0.0480 (7)	0.0437 (7)	0.0533 (8)	0.0039 (7)	0.0059 (6)	0.0130 (7)
C13	0.0394 (9)	0.0440 (10)	0.0472 (10)	−0.0087 (9)	0.0022 (7)	0.0005 (9)
C14	0.0479 (10)	0.0664 (14)	0.0444 (10)	−0.0136 (10)	0.0075 (8)	0.0057 (10)
C15	0.0486 (10)	0.0630 (13)	0.0374 (10)	0.0065 (10)	0.0088 (8)	0.0042 (10)
O3	0.0546 (8)	0.0639 (9)	0.0369 (7)	−0.0138 (7)	0.0028 (6)	0.0009 (7)
O4	0.0871 (11)	0.0960 (14)	0.0462 (8)	−0.0236 (12)	0.0156 (8)	0.0069 (10)
C16	0.0489 (10)	0.0554 (12)	0.0395 (10)	0.0059 (10)	0.0048 (8)	−0.0042 (9)
C17	0.0584 (12)	0.0580 (13)	0.0451 (11)	0.0031 (11)	0.0095 (9)	−0.0044 (10)
C18	0.0831 (16)	0.0657 (16)	0.0636 (15)	−0.0101 (14)	0.0185 (12)	−0.0084 (13)
C19	0.0741 (16)	0.088 (2)	0.0684 (16)	−0.0170 (16)	0.0097 (13)	−0.0245 (16)
C20	0.0841 (17)	0.106 (2)	0.0478 (13)	−0.0097 (18)	−0.0033 (12)	−0.0182 (16)
C21	0.0778 (15)	0.0861 (19)	0.0405 (11)	−0.0056 (15)	0.0037 (10)	0.0000 (12)
C22	0.0349 (8)	0.0389 (10)	0.0408 (9)	0.0000 (8)	0.0009 (7)	−0.0039 (8)
O5	0.0380 (6)	0.0471 (8)	0.0482 (7)	0.0065 (6)	−0.0011 (5)	−0.0111 (6)
O6	0.0558 (8)	0.0545 (9)	0.0761 (10)	−0.0015 (8)	0.0079 (7)	−0.0252 (9)
C23	0.0429 (9)	0.0365 (10)	0.0474 (10)	−0.0012 (8)	0.0098 (8)	−0.0019 (9)
C24	0.0411 (9)	0.0481 (11)	0.0443 (10)	0.0030 (9)	0.0116 (8)	0.0094 (9)
C25	0.0582 (12)	0.0618 (14)	0.0616 (13)	0.0162 (12)	0.0215 (10)	0.0190 (12)
C26	0.0622 (15)	0.095 (2)	0.0780 (17)	0.0330 (16)	0.0267 (13)	0.0387 (17)
C27	0.0468 (13)	0.123 (3)	0.0780 (18)	0.0130 (17)	0.0044 (12)	0.048 (2)
C28	0.0629 (15)	0.095 (2)	0.0812 (18)	−0.0118 (16)	−0.0149 (13)	0.0114 (17)
C29	0.0522 (12)	0.0651 (15)	0.0647 (14)	−0.0008 (12)	−0.0019 (10)	0.0026 (12)
C30	0.0417 (9)	0.0388 (10)	0.0385 (9)	0.0017 (8)	0.0040 (7)	−0.0005 (8)
O7	0.0436 (7)	0.0520 (8)	0.0394 (7)	−0.0014 (6)	0.0051 (5)	−0.0093 (6)
O8	0.0559 (9)	0.0925 (13)	0.0672 (10)	−0.0132 (10)	0.0234 (7)	−0.0204 (10)
C31	0.0494 (11)	0.0585 (13)	0.0404 (10)	0.0063 (10)	0.0076 (8)	−0.0048 (9)
C32	0.0521 (11)	0.0568 (12)	0.0355 (9)	0.0057 (10)	0.0034 (8)	−0.0033 (9)
C33	0.0683 (14)	0.0726 (16)	0.0567 (13)	−0.0108 (13)	0.0225 (11)	−0.0148 (13)
C34	0.0811 (17)	0.089 (2)	0.0725 (16)	−0.0296 (17)	0.0256 (14)	−0.0197 (16)
C35	0.102 (2)	0.0711 (18)	0.0631 (15)	−0.0160 (17)	0.0086 (14)	−0.0229 (14)
C36	0.0784 (17)	0.096 (2)	0.0663 (15)	0.0148 (16)	0.0043 (13)	−0.0357 (16)
C37	0.0544 (12)	0.0889 (19)	0.0549 (13)	0.0114 (13)	0.0043 (10)	−0.0217 (13)

### Geometric parameters (Å, º)

**Table:**

O1—C1	1.220 (2)	C17—C18	1.383 (3)
C1—N2	1.391 (2)	C17—H17	0.9300
C1—C2	1.455 (3)	C18—C19	1.379 (4)
C2—C7	1.394 (3)	C18—H18	0.9300
C2—C3	1.398 (3)	C19—C20	1.374 (4)
C3—C4	1.376 (3)	C19—H19	0.9300
C3—H3	0.9300	C20—C21	1.373 (4)
C4—C5	1.384 (4)	C20—H20	0.9300
C4—H4	0.9300	C21—H21	0.9300
C5—C6	1.357 (4)	C22—O5	1.439 (2)
C5—H5	0.9300	C22—C30	1.517 (2)
C6—C7	1.397 (3)	C22—H22	0.9800
C6—H6	0.9300	O5—C23	1.348 (2)
C7—N1	1.390 (3)	O6—C23	1.196 (2)
N1—C8	1.280 (3)	C23—C24	1.485 (3)
N2—C8	1.372 (2)	C24—C29	1.377 (3)
N2—C9	1.462 (3)	C24—C25	1.388 (3)
C8—H8	0.9300	C25—C26	1.383 (3)
C9—C10	1.500 (3)	C25—H25	0.9300
C9—H9A	0.9700	C26—C27	1.356 (5)
C9—H9B	0.9700	C26—H26	0.9300
C10—N3	1.349 (2)	C27—C28	1.374 (5)
C10—C11	1.356 (3)	C27—H27	0.9300
C11—N5	1.348 (2)	C28—C29	1.385 (3)
C11—H11	0.9300	C28—H28	0.9300
N3—N4	1.308 (2)	C29—H29	0.9300
N4—N5	1.343 (2)	C30—O7	1.439 (2)
N5—C12	1.463 (2)	C30—H30	0.9800
C12—O2	1.406 (2)	O7—C31	1.351 (2)
C12—C30	1.529 (2)	O8—C31	1.207 (2)
C12—H12	0.9800	C31—C32	1.480 (3)
O2—C13	1.442 (2)	C32—C33	1.380 (3)
C13—C14	1.496 (3)	C32—C37	1.387 (3)
C13—C22	1.524 (3)	C33—C34	1.385 (4)
C13—H13	0.9800	C33—H33	0.9300
C14—O3	1.437 (3)	C34—C35	1.376 (4)
C14—H14A	0.9700	C34—H34	0.9300
C14—H14B	0.9700	C35—C36	1.362 (4)
C15—O4	1.204 (3)	C35—H35	0.9300
C15—O3	1.343 (2)	C36—C37	1.365 (4)
C15—C16	1.479 (3)	C36—H36	0.9300
C16—C17	1.381 (3)	C37—H37	0.9300
C16—C21	1.395 (3)		
			
O1—C1—N2	120.65 (17)	C16—C17—C18	120.0 (2)
O1—C1—C2	125.40 (18)	C16—C17—H17	120.0
N2—C1—C2	113.95 (16)	C18—C17—H17	120.0
C7—C2—C3	120.29 (19)	C19—C18—C17	120.0 (3)
C7—C2—C1	119.44 (18)	C19—C18—H18	120.0
C3—C2—C1	120.26 (18)	C17—C18—H18	120.0
C4—C3—C2	119.2 (2)	C20—C19—C18	120.3 (3)
C4—C3—H3	120.4	C20—C19—H19	119.9
C2—C3—H3	120.4	C18—C19—H19	119.9
C3—C4—C5	120.2 (2)	C21—C20—C19	120.2 (2)
C3—C4—H4	119.9	C21—C20—H20	119.9
C5—C4—H4	119.9	C19—C20—H20	119.9
C6—C5—C4	121.2 (2)	C20—C21—C16	120.1 (3)
C6—C5—H5	119.4	C20—C21—H21	120.0
C4—C5—H5	119.4	C16—C21—H21	120.0
C5—C6—C7	120.0 (2)	O5—C22—C30	110.78 (13)
C5—C6—H6	120.0	O5—C22—C13	111.78 (15)
C7—C6—H6	120.0	C30—C22—C13	103.22 (16)
N1—C7—C2	122.38 (18)	O5—C22—H22	110.3
N1—C7—C6	118.5 (2)	C30—C22—H22	110.3
C2—C7—C6	119.1 (2)	C13—C22—H22	110.3
C8—N1—C7	116.71 (17)	C23—O5—C22	116.25 (14)
C8—N2—C1	121.92 (17)	O6—C23—O5	122.97 (17)
C8—N2—C9	119.88 (18)	O6—C23—C24	124.52 (18)
C1—N2—C9	118.16 (16)	O5—C23—C24	112.50 (16)
N1—C8—N2	125.5 (2)	C29—C24—C25	119.8 (2)
N1—C8—H8	117.2	C29—C24—C23	122.45 (19)
N2—C8—H8	117.2	C25—C24—C23	117.7 (2)
N2—C9—C10	112.83 (17)	C26—C25—C24	119.7 (3)
N2—C9—H9A	109.0	C26—C25—H25	120.2
C10—C9—H9A	109.0	C24—C25—H25	120.2
N2—C9—H9B	109.0	C27—C26—C25	120.3 (3)
C10—C9—H9B	109.0	C27—C26—H26	119.9
H9A—C9—H9B	107.8	C25—C26—H26	119.9
N3—C10—C11	108.93 (16)	C26—C27—C28	120.6 (2)
N3—C10—C9	119.82 (17)	C26—C27—H27	119.7
C11—C10—C9	131.24 (18)	C28—C27—H27	119.7
N5—C11—C10	104.50 (17)	C27—C28—C29	119.9 (3)
N5—C11—H11	127.7	C27—C28—H28	120.1
C10—C11—H11	127.7	C29—C28—H28	120.1
N4—N3—C10	108.91 (16)	C24—C29—C28	119.8 (3)
N3—N4—N5	107.01 (15)	C24—C29—H29	120.1
N4—N5—C11	110.65 (16)	C28—C29—H29	120.1
N4—N5—C12	122.23 (15)	O7—C30—C22	108.31 (14)
C11—N5—C12	126.70 (16)	O7—C30—C12	108.15 (15)
O2—C12—N5	110.41 (14)	C22—C30—C12	100.90 (14)
O2—C12—C30	106.29 (14)	O7—C30—H30	112.9
N5—C12—C30	111.07 (15)	C22—C30—H30	112.9
O2—C12—H12	109.7	C12—C30—H30	112.9
N5—C12—H12	109.7	C31—O7—C30	115.70 (15)
C30—C12—H12	109.7	O8—C31—O7	122.9 (2)
C12—O2—C13	110.98 (14)	O8—C31—C32	124.97 (19)
O2—C13—C14	108.78 (15)	O7—C31—C32	112.15 (17)
O2—C13—C22	104.75 (14)	C33—C32—C37	119.3 (2)
C14—C13—C22	118.86 (19)	C33—C32—C31	122.55 (19)
O2—C13—H13	108.0	C37—C32—C31	118.14 (19)
C14—C13—H13	108.0	C32—C33—C34	119.6 (2)
C22—C13—H13	108.0	C32—C33—H33	120.2
O3—C14—C13	110.08 (15)	C34—C33—H33	120.2
O3—C14—H14A	109.6	C35—C34—C33	120.2 (2)
C13—C14—H14A	109.6	C35—C34—H34	119.9
O3—C14—H14B	109.6	C33—C34—H34	119.9
C13—C14—H14B	109.6	C36—C35—C34	120.0 (3)
H14A—C14—H14B	108.2	C36—C35—H35	120.0
O4—C15—O3	122.2 (2)	C34—C35—H35	120.0
O4—C15—C16	124.62 (18)	C35—C36—C37	120.5 (2)
O3—C15—C16	113.16 (17)	C35—C36—H36	119.7
C15—O3—C14	115.17 (16)	C37—C36—H36	119.7
C17—C16—C21	119.5 (2)	C36—C37—C32	120.4 (2)
C17—C16—C15	122.56 (17)	C36—C37—H37	119.8
C21—C16—C15	117.9 (2)	C32—C37—H37	119.8
			
O1—C1—C2—C7	−179.64 (19)	O3—C15—C16—C21	−170.2 (2)
N2—C1—C2—C7	0.4 (2)	C21—C16—C17—C18	−0.8 (3)
O1—C1—C2—C3	0.9 (3)	C15—C16—C17—C18	177.3 (2)
N2—C1—C2—C3	−178.99 (16)	C16—C17—C18—C19	1.0 (4)
C7—C2—C3—C4	−0.7 (3)	C17—C18—C19—C20	−0.5 (4)
C1—C2—C3—C4	178.7 (2)	C18—C19—C20—C21	−0.3 (5)
C2—C3—C4—C5	0.2 (4)	C19—C20—C21—C16	0.5 (4)
C3—C4—C5—C6	0.2 (4)	C17—C16—C21—C20	0.0 (4)
C4—C5—C6—C7	−0.1 (4)	C15—C16—C21—C20	−178.1 (2)
C3—C2—C7—N1	−178.85 (19)	O2—C13—C22—O5	147.77 (14)
C1—C2—C7—N1	1.7 (3)	C14—C13—C22—O5	−90.54 (19)
C3—C2—C7—C6	0.8 (3)	O2—C13—C22—C30	28.67 (18)
C1—C2—C7—C6	−178.6 (2)	C14—C13—C22—C30	150.35 (16)
C5—C6—C7—N1	179.3 (2)	C30—C22—O5—C23	−148.57 (16)
C5—C6—C7—C2	−0.4 (4)	C13—C22—O5—C23	96.90 (19)
C2—C7—N1—C8	−2.3 (3)	C22—O5—C23—O6	−1.7 (3)
C6—C7—N1—C8	178.0 (2)	C22—O5—C23—C24	178.91 (16)
O1—C1—N2—C8	178.11 (19)	O6—C23—C24—C29	−162.5 (2)
C2—C1—N2—C8	−2.0 (2)	O5—C23—C24—C29	16.9 (3)
O1—C1—N2—C9	−4.4 (3)	O6—C23—C24—C25	14.6 (3)
C2—C1—N2—C9	175.55 (15)	O5—C23—C24—C25	−166.00 (16)
C7—N1—C8—N2	0.8 (3)	C29—C24—C25—C26	0.7 (3)
C1—N2—C8—N1	1.5 (3)	C23—C24—C25—C26	−176.40 (19)
C9—N2—C8—N1	−176.0 (2)	C24—C25—C26—C27	0.4 (3)
C8—N2—C9—C10	−95.6 (2)	C25—C26—C27—C28	−1.3 (4)
C1—N2—C9—C10	86.8 (2)	C26—C27—C28—C29	1.0 (4)
N2—C9—C10—N3	152.32 (18)	C25—C24—C29—C28	−1.0 (3)
N2—C9—C10—C11	−28.9 (3)	C23—C24—C29—C28	176.0 (2)
N3—C10—C11—N5	−0.2 (2)	C27—C28—C29—C24	0.1 (4)
C9—C10—C11—N5	−179.07 (19)	O5—C22—C30—O7	−42.7 (2)
C11—C10—N3—N4	0.2 (2)	C13—C22—C30—O7	77.08 (16)
C9—C10—N3—N4	179.22 (17)	O5—C22—C30—C12	−156.17 (15)
C10—N3—N4—N5	−0.1 (2)	C13—C22—C30—C12	−36.37 (17)
N3—N4—N5—C11	0.0 (2)	O2—C12—C30—O7	−81.39 (17)
N3—N4—N5—C12	173.02 (15)	N5—C12—C30—O7	158.48 (14)
C10—C11—N5—N4	0.1 (2)	O2—C12—C30—C22	32.19 (18)
C10—C11—N5—C12	−172.54 (17)	N5—C12—C30—C22	−87.95 (17)
N4—N5—C12—O2	−101.9 (2)	C22—C30—O7—C31	176.14 (16)
C11—N5—C12—O2	70.0 (2)	C12—C30—O7—C31	−75.31 (19)
N4—N5—C12—C30	15.8 (2)	C30—O7—C31—O8	0.3 (3)
C11—N5—C12—C30	−172.38 (17)	C30—O7—C31—C32	178.77 (15)
N5—C12—O2—C13	105.43 (16)	O8—C31—C32—C33	−170.3 (2)
C30—C12—O2—C13	−15.1 (2)	O7—C31—C32—C33	11.3 (3)
C12—O2—C13—C14	−136.61 (18)	O8—C31—C32—C37	10.9 (3)
C12—O2—C13—C22	−8.5 (2)	O7—C31—C32—C37	−167.5 (2)
O2—C13—C14—O3	60.5 (2)	C37—C32—C33—C34	0.1 (4)
C22—C13—C14—O3	−59.1 (2)	C31—C32—C33—C34	−178.7 (2)
O4—C15—O3—C14	−1.7 (3)	C32—C33—C34—C35	0.5 (4)
C16—C15—O3—C14	179.56 (18)	C33—C34—C35—C36	−0.5 (5)
C13—C14—O3—C15	−157.97 (19)	C34—C35—C36—C37	−0.1 (5)
O4—C15—C16—C17	−167.0 (2)	C35—C36—C37—C32	0.7 (4)
O3—C15—C16—C17	11.7 (3)	C33—C32—C37—C36	−0.7 (4)
O4—C15—C16—C21	11.1 (3)	C31—C32—C37—C36	178.2 (2)

### Hydrogen-bond geometry (Å, º)

**Table:**

*D*—H···*A*	*D*—H	H···*A*	*D*···*A*	*D*—H···*A*
C3—H3···O6^i^	0.93	2.57	3.483 (3)	168
C13—H13···O6^ii^	0.98	2.36	3.293 (3)	159
C6—H6···N1^iii^	0.93	2.62	3.390 (3)	141

Symmetry codes: (i) *x*+1, *y*−1, *z*; (ii) *x*, *y*−1, *z*; (iii) −*x*+3, *y*−1/2, −*z*+2.
